# Insulitis in the pancreas of non-diabetic organ donors under age 25 years with multiple circulating autoantibodies against islet cell antigens

**DOI:** 10.1007/s00428-021-03055-z

**Published:** 2021-02-16

**Authors:** Silke Smeets, Diedert Luc De Paep, Geert Stangé, Katrijn Verhaeghen, Bart Van der Auwera, Bart Keymeulen, Ilse Weets, Zhidong Ling, Peter in’t Veld, Frans Gorus

**Affiliations:** 1 Diabetes Research Center (DRC), Vrije Universiteit Brussel (VUB), Brussels, Belgium; 2 Beta Cell Bank, UZ Brussel, Brussels, Belgium; 3 Department of Surgery, UZ Brussel, Brussels, Belgium; 4 Clinical Biology, UZ Brussel, Brussels, Belgium

**Keywords:** Insulitis, Autoantibodies, Type 1 diabetes, Islets, Beta cells, HLA-DQ, HLA class I

## Abstract

Autoantibodies against islet cell antigens are routinely used to identify subjects at increased risk of symptomatic type 1 diabetes, but their relation to the intra-islet pathogenetic process that leads to positivity for these markers is poorly understood. We screened 556 non-diabetic organ donors (3 months to 24 years) for five different autoantibodies and found positivity in 27 subjects, 25 single- and two double autoantibody-positive donors. Histopathological screening of pancreatic tissue samples showed lesion characteristic for recent-onset type 1 diabetes in the two organ donors with a high-risk profile, due to their positivity for multiple autoantibodies and HLA-inferred risk. Inflammatory infiltrates (insulitis) were found in a small fraction of islets (<5%) and consisted predominantly of CD3+CD8+ T-cells. Islets with insulitis were found in close proximity to islets devoid of insulin-positivity; such pseudo-atrophic islets were present in multiple small foci scattered throughout the pancreatic tissue or were found to be distributed with a lobular pattern. Relative beta cell area in both single and multiple autoantibody-positive donors was comparable to that in autoantibody-negative controls. In conclusion, in organ donors under age 25 years, insulitis and pseudo-atrophic islets were restricted to multiple autoantibody-positive individuals allegedly at high risk of developing symptomatic type 1 diabetes, in line with reports in older age groups. These observations may give further insight into the early pathogenetic events that may culminate in clinically overt disease.

**Supplementary Information:**

The online version contains supplementary material available at 10.1007/s00428-021-03055-z.

## Introduction

Insulitis is an inflammatory lesion of the islets of Langerhans characteristic for patients with recent-onset type 1 diabetes. The infiltrate predominantly consists of CD8+ T-lymphocytes of which a fraction is thought to mediate beta cell specific cytotoxicity [1–4]. It is now widely accepted that clinical onset of type 1 diabetes is preceded by a usually long hidden disease process as judged by the appearance of circulating antibodies against beta cell antigens including islet cell cytoplasm (ICA) [5], insulin (IAA) [6], glutamic acid decarboxylase (GADA) [7], insulinoma-associated protein 2 (IA-2A) [8], and zinc transporter 8 (ZnT8A) [9], months to decades before diagnosis [10, 11]. These autoantibodies tend to develop sequentially rather than simultaneously [12], with IA-2A and ZnT8A usually appearing closer to clinical onset [13]. Both in children and in adults up to 40 years of age, presence of at least two molecularly defined autoantibodies confers 90% risk of developing clinical onset within 20 years, and is now termed asymptomatic type 1 diabetes [10, 11, 14]. However, the relationship between the development of autoantibodies and the development of histopathological hallmarks of type 1 diabetes during the natural history of asymptomatic disease is poorly understood. In a now classical model for the development of type 1 diabetes, autoantibodies are considered to be indicators for ongoing autoimmune beta cell destruction in genetically susceptible individuals, triggered, and promoted by as yet unidentified environmental factors [15, 16]. At clinical onset, functional beta cell mass has dropped to 10–40% of normal [17], but the timing and kinetics of its decline during the asymptomatic phase remain a matter of debate [15, 16]. The classical model remains to a large extent hypothetical since firm evidence supporting a causal link between insulitis and circulating autoantibodies is lacking. In previous histopathological studies, donor pancreata from autoantibody-positive non-diabetic organ donors—used as a model for at risk individuals [3, 4, 18–22]—showed no evidence of decreased relative beta cell area [4, 18, 20, 21]. Only four autoantibody-positive individuals in total were identified with insulitis according to international consensus criteria [4, 18], half of them in subjects >45 years of age, an age group that is substantially older than the group of 10–14 years in which the incidence of type 1 diabetes is at its highest level [1]. Insight into early histopathological changes in islet tissue in autoantibody-positive individuals under the age of 25 years is therefore limited and formed the rationale for studying a large cohort of non-diabetic organ donors below the age of 25 years. We compared histopathological changes in islet tissue from autoantibody-positive organ donors to a matched control group of autoantibody-negative organ donors from the same cohort.

## Methods

### Collection of pancreatic tissue

Human donor pancreases were obtained in the context of our islet transplantation program. A single tissue sample (~0.5 cm^3^) was taken from the pancreatic body of cold-preserved deceased donor organs provided by Eurotransplant Foundation (Leiden, The Netherlands), fixed in phosphate-buffered 4% formaldehyde or Bouin’s fixative, and embedded in paraffin. Some donor organs were eventually not processed for islet isolation, and in these cases, multiple sample blocks from the resected organ were available for study. Biopsies were taken as part of a quality control procedure that was approved by the local ethics committee (approval number BUN143201941720) and stored in a registered Biobank (Diabetes Biobank Brussels, approval number local ethics committee BUN143201524128). Between 1989 and 2019, 556 organs met the following inclusion criteria: donor age below 25 years, availability of minimal clinical data, a serum sample for islet cell autoantibody assays, and a formalin or Bouin fixed tissue sample. Diabetes was an exclusion criterion for organ procurement. Within the group of 556 organ donors, a total of 27 were found to be positive for one or more autoantibodies directed against islet cell antigens. Pancreas tissue from this subgroup was screened for histopathological changes as described below, and the results were compared to a control group of 27 autoantibody-negative organ donors obtained from the same cohort and matched for age, sex, and BMI. There is no overlap whatsoever between the pancreata from young donors with or without autoantibodies studied here (<25 years) and the pancreata from a previous report on older donors (≥25 years) [18].

### Autoantibodies and genetic risk markers

Serum samples were tested for the presence of ICA, IA-2A, GADA, and IAA [23], and when positive for at least one of those markers, also evaluated for ZnT8A [24]. ICA were assessed by indirect immunofluorescence and endpoint titers expressed as JDF units. IA-2A, GADA, IAA, and ZnT8A were measured by liquid-phase radiobinding assay and expressed as either percent tracer bound in hemolysis-free sera (IAA, ZnT8A) or WHO U/mL (IA-2A, GADA) [23, 24]. Cut-off values for autoantibody positivity were calculated as 99th percentile of autoantibody levels in 790 non-diabetic controls after exclusion of outlying values (≥12 JDF units for ICA, ≥1.4 WHO U/ml for IA-2A, ≥23 WHO U/ml for GADA, ≥0.6% tracer binding for IAA, and ≥1.28% (0–14 years) or ≥1.02% tracer binding (>14 years) for ZnT8A. The autoantibody assays were validated in successive international proficiency testing programs (DASP, IASP); all positive results were confirmed in a separate subsequent assay [13, 23]. Whole blood was haplotyped for DNA polymorphisms at the *HLA-DQA1* and *DQB1* gene loci and DQ-associated risk stratified as reported previously [25]. The presence of HLA class I alleles conferring susceptibility for type 1 diabetes independently of HLA class II inferred risk was deduced from information provided by Eurotransplant.

### Screening for insulitis and pseudo-atrophic islets

Sections were processed and stained for CD45 and synaptophysin for the detection of insulitis and for insulin and glucagon to screen for pseudo-atrophic (insulin-deficient) islets as previously described [18] (see also Supplementary methods). We screened at least 1 cm^2^, a minimum of 100 islets, and an average of 280±43 (±SEM) islets per donor organ. Each section was analyzed by two observers, who were blinded as to the identity of the samples. When insulitis or pseudo-atrophic islets were found, the available blocks were sectioned completely for analysis of every twentieth section. The diagnosis of insulitis was made according to the JDRF-nPOD consensus criteria [26]. Briefly, insulitis is diagnosed when ≥3 islets with ≥15 CD45+ cells are found in an organ that also contains pseudo-atrophic islets. We considered clusters of ≥5 endocrine cells as islets and called them “insulitic” when the threshold of ≥15 CD45+ cells was exceeded and “pseudo-atrophic” when the islets were devoid of insulin immunoreactive cells.

### Characterization of leucocytic infiltrates

Leucocytic infiltrates were immunophenotyped on paraffin sections using immunofluorescent staining for CD3, CD45, CD4, CD8, CD20, and CD68 as previously described [18] (see also Supplementary methods). Leucocytic infiltrates were examined with a fluorescence microscope Nikon Eclipse 80i (Nikon BeLux; Brussels; Belgium) equipped with an Orca AG camera (Hamamatsu; Herrsching am Ammersee, Germany) and imaging software NIS elements AR (Nikon BeLux). Negative controls included the omission of the primary antibody and positive controls used paraffin-embedded human tonsil.

### Quantification of relative beta cell area and beta cell proliferation

For quantification of the relative beta cell area, paraffin sections were stained for insulin. The relative insulin-positive cell area was measured as previously published [18] (see also Supplementary methods).

Beta cell replication was quantified using double staining for insulin and Ki67 (Supplementary methods). A minimum of 1000 insulin-positive cells per donor were analyzed for Ki67 positivity.

### Statistical analysis

Differences between groups were analyzed by an unpaired *t* test or Mann-Whitney test (two-tailed) for, respectively, normal distributions and non-normal distributions. Spearman correlation was used to examine the relationship between different parameters. All statistical tests were performed using GraphPad Prism 8 (GraphPad Software; San Diego; CA; USA) and considered significant at *p*<0.05.

## Results

### Screening for autoantibodies

The study population consisted of 556 organ donors <25 years with a median age of 19 years (range 3 months to 24 years) (Table 1). Serum samples were screened for the presence of autoantibodies resulting in a total of 27 (4.9%) positive donors. Their clinical characteristics did not differ significantly from that of the entire study population (Table 1). Twenty-five individuals were positive for a single autoantibody, with a predominance of GADA (Table S1). Two donors were positive for two autoantibody markers, one for ICA and GADA (DBB-3504), the other for IA-2A and ZnTA8 (DBB-A096) (Table S2).

**Table 1:** Clinical characteristics of autoantibody-positive organ donors, matched controls, and total study population

Characteristics	Autoantibody-positive donors	Matched controls	All donors
Number	27	27	556
Donor age (yrs, median (IQR))	19 (16–20)	19 (16–20)	18 (13–20)
Age range (yrs)	2–24	1–25	3 months–24
BMI (kg/m^2^, median (IQR))	21.9 (19.5–23.7)	22.2 (19.6–23.0)	21.2 (18.7–23.1)
Sex			
Female (*n*)	6	6	172
Male (*n*)	21	21	384
Stay in ICU (days, median (IQR))	2 (1–5)	2 (1–5)	2 (1–4)

From 22 of the 27 autoantibody-positive donors, a segmental pancreas was received, but from five individuals, the intact organ was available for study. The mean pancreatic weight of intact organs was 68.4±25.3g (±SD), which was not significantly different from that in ten controls matched for age, sex, and BMI (71.7±26.6g).

### Beta cell area and replication

**Fig. 1:**
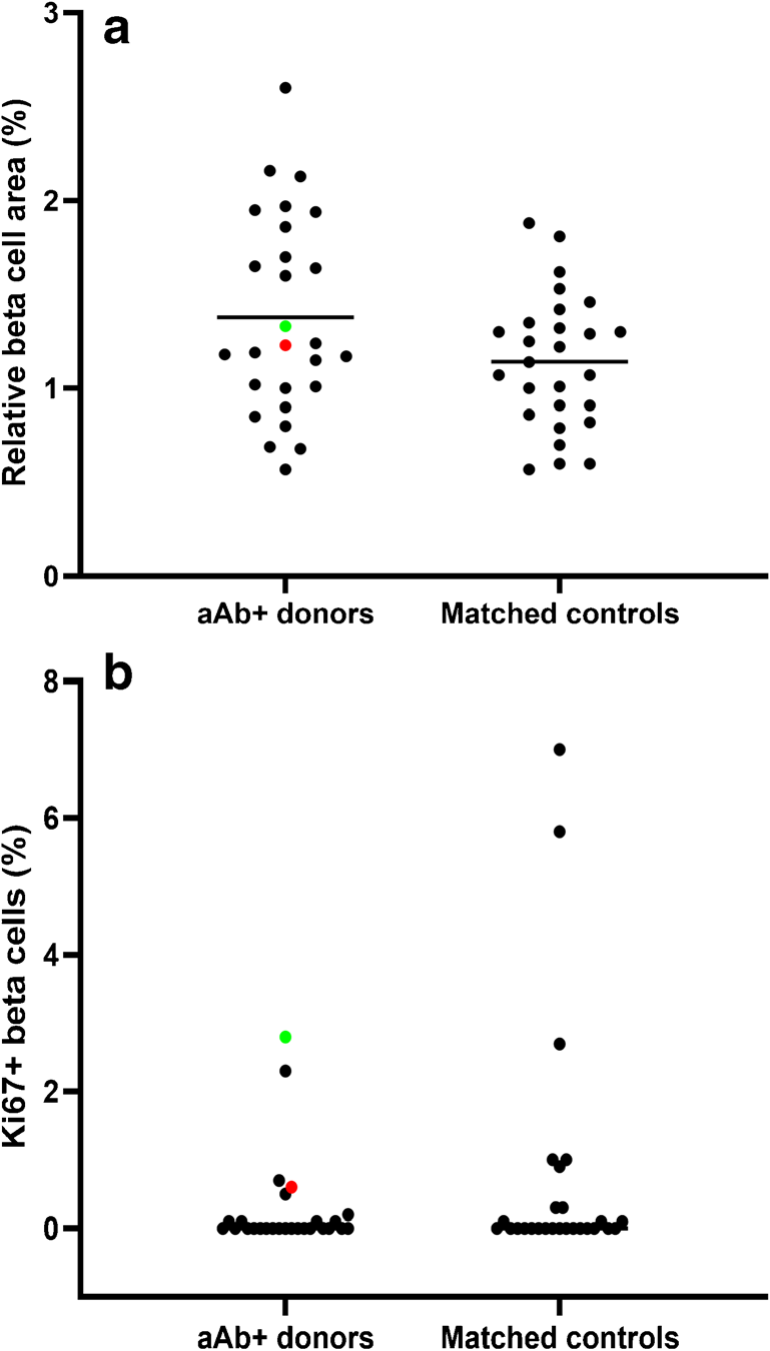
Relative beta cell area (**a**) and the percentage of replicating beta cells (**b**) in the pancreas of autoantibody-positive organ donors (*n*=27) and matched controls (*n*=27). The two donors with multiple autoantibodies and insulitis are indicated with a green dot (DBB-A096) and a red dot (DBB-3504). Results are expressed as mean (**a**) or median (**b**) with individual data points. No significant difference was found between both study groups using an unpaired Student *t* test (**a**) or an unpaired Mann-Whitney test (**b**) at the .05* level

The relative beta cell area was measured in all 27 autoantibody-positive donors and an equal number of autoantibody-negative controls matched for age, sex, and BMI (Table 1). The mean relative beta cell area in autoantibody-positive donors was 1.38±0.53% (±SD), which was not significantly different from that in the matched controls (1.14±0.36%) (Fig. 1a). No significant difference could be found at the level of beta cell replication between the autoantibody-positive organ donors (median (IQR) 0.0% (0.0–0.1%)) and the matched controls (0.0% (0.0–0.3%)); some donors (both autoantibody-positive and -negative) showed high replication levels, up to 7% (Fig. 1b), corresponding to levels seen in earlier studies in donors characterized by a longer duration of stay in intensive care [52]. No correlation could be found between beta cell area and age, and between beta cell replication and age (data not shown).

### Screening for insulitis

Pancreas biopsies from all 27 autoantibody-positive donors and an equal number of autoantibody-negative-matched controls were screened for the presence of insulitis as defined by the international consensus criteria. Sections were stained for synaptophysin and CD45, to detect and quantify the presence of leucocytic infiltrates. They were also stained for insulin and glucagon to detect insulin-deficient pseudo-atrophic islets. None of the 25 single autoantibody-positive donors, or the 27 controls, showed evidence of insulitis. However, the two donor organs with multiple autoantibodies were both diagnosed with insulitis and showed multiple insulitic islets together with focal areas rich in pseudo-atrophic islets.

**Fig. 2:**
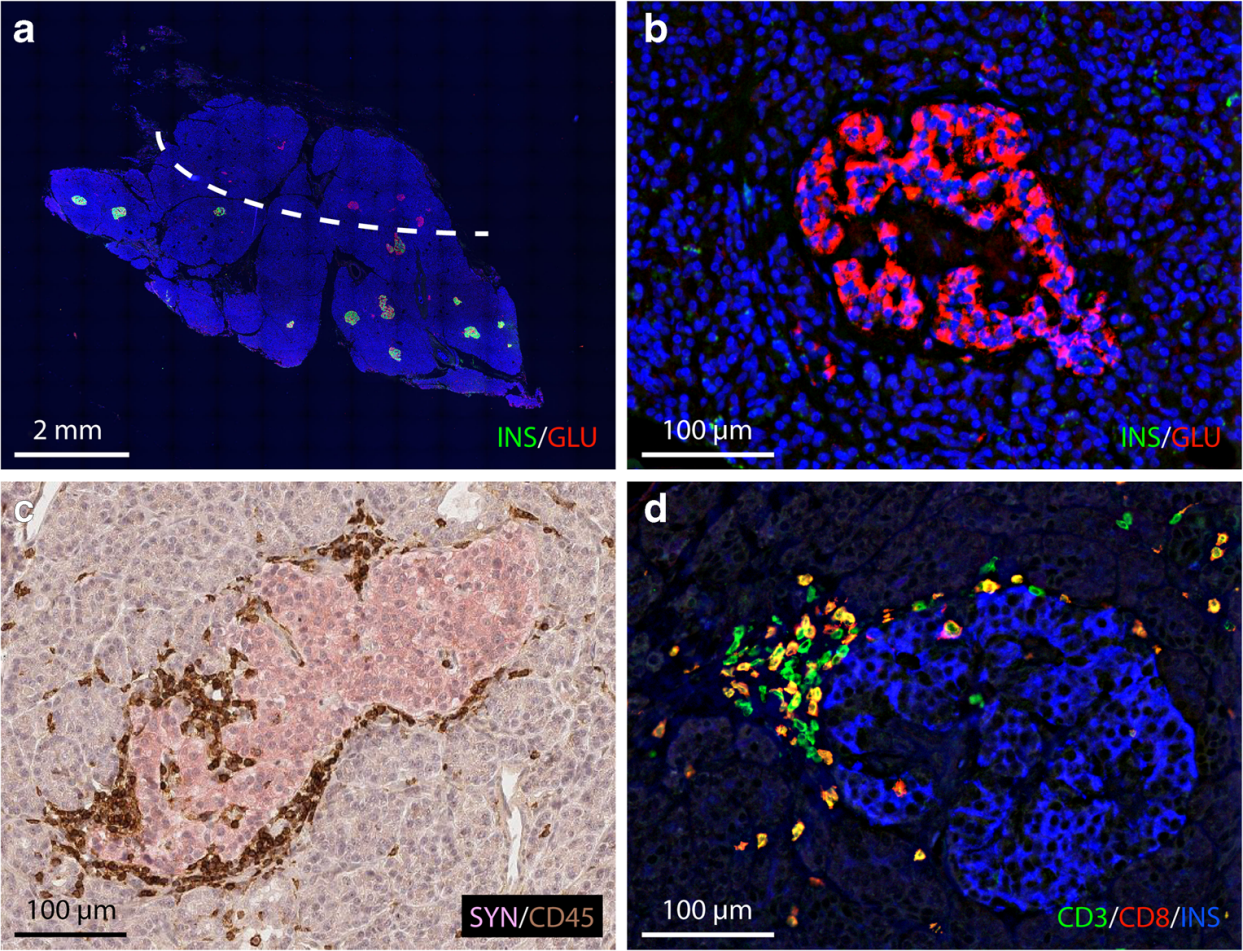
Immunofluorescent staining of pancreas sections from a 22-year-old male organ donor positive for GADA and ICA autoantibodies (DBB-3504). Staining for insulin (green) and glucagon (red) reveals a lobular area (above the dotted line) (**a**) containing pseudo-atrophic islets devoid of insulin (**b**). Immunostaining for the leucocytic marker CD45 (brown) in combination with the pan-endocrine marker synaptophysin (red) showing peri-insulitis (**c**). Staining for CD3 (green), CD8 (red), and insulin (blue) shows an insulitic lesion consisting of a cluster of predominantly lymphocytic cells in the islet periphery (**d**)

Donor DBB-3504 involved a 22-year-old male donor who died of polytrauma, 11 days after admission to the intensive care unit (ICU). He was tested positive for two autoantibodies (ICA, 400 JDF units; GADA, 29655 WHO units/ml) and had a protective HLA-DQA1/DQB1 genotype (*02-02:01/03-03:01*) and a HLA class I risk allele (*A24*) [27, 28]. The entire donor organ was received with a total weight of 53g and processed for islet isolation and transplantation. A single tissue sample (approx. 0.5 cm^3^) from the body of the gland was available for histopathological study. Low magnification images of tissue sections double-stained for insulin and glucagon showed both normal and pseudo-atrophic islets devoid of insulin-immunoreactivity, with a lobular distribution of the latter islet type. In total, 58.2% of all islets analyzed were categorized as pseudo-atrophic (Fig. 2a–b). Double staining for CD45 and synaptophysin showed insulitis in 3.8% of the islets, with the infiltrate being mainly (74%) present in the islet periphery (peri-insulitis) (Fig. 2c). The infiltrate consisted predominantly of CD3+CD8+-positive T-lymphocytes (76% of all CD3+ cells) (Fig. 2d). In addition, small numbers CD3+CD4+ T-lymphocytes and CD20+ B-lymphocytes were present. The infiltrates showed a CD20Lo insulitic profile, with a CD20/CD4 ratio <1.0 [29]. A more extensive characterization of the infiltrating cells was precluded by the small size of the infiltrates. All insulitic islets still contained insulin-positive beta cells. A beta cell replication rate of 0.6% was found in this donor, in addition to a relative beta cell area of 1.23% (Table 2).

**Table 2:** Autoantibody-positive pancreas donors with insulitis

Characteristics	Donor ID	
	3504	A096
Age (years)/sex	22/M	17/M
Type of autoantibody-positivity	ICA + GADA	IA-2A + ZnT8A
Total number of islets investigated	612	18,242
% of islets with insulitis	3.8	0.2
% of pseudo-atrophic islets	58.2	7.7
Relative beta cell area (%)	1.23	1.33
Replicating beta cells (%)	0.6	2.8

**Fig. 3:**
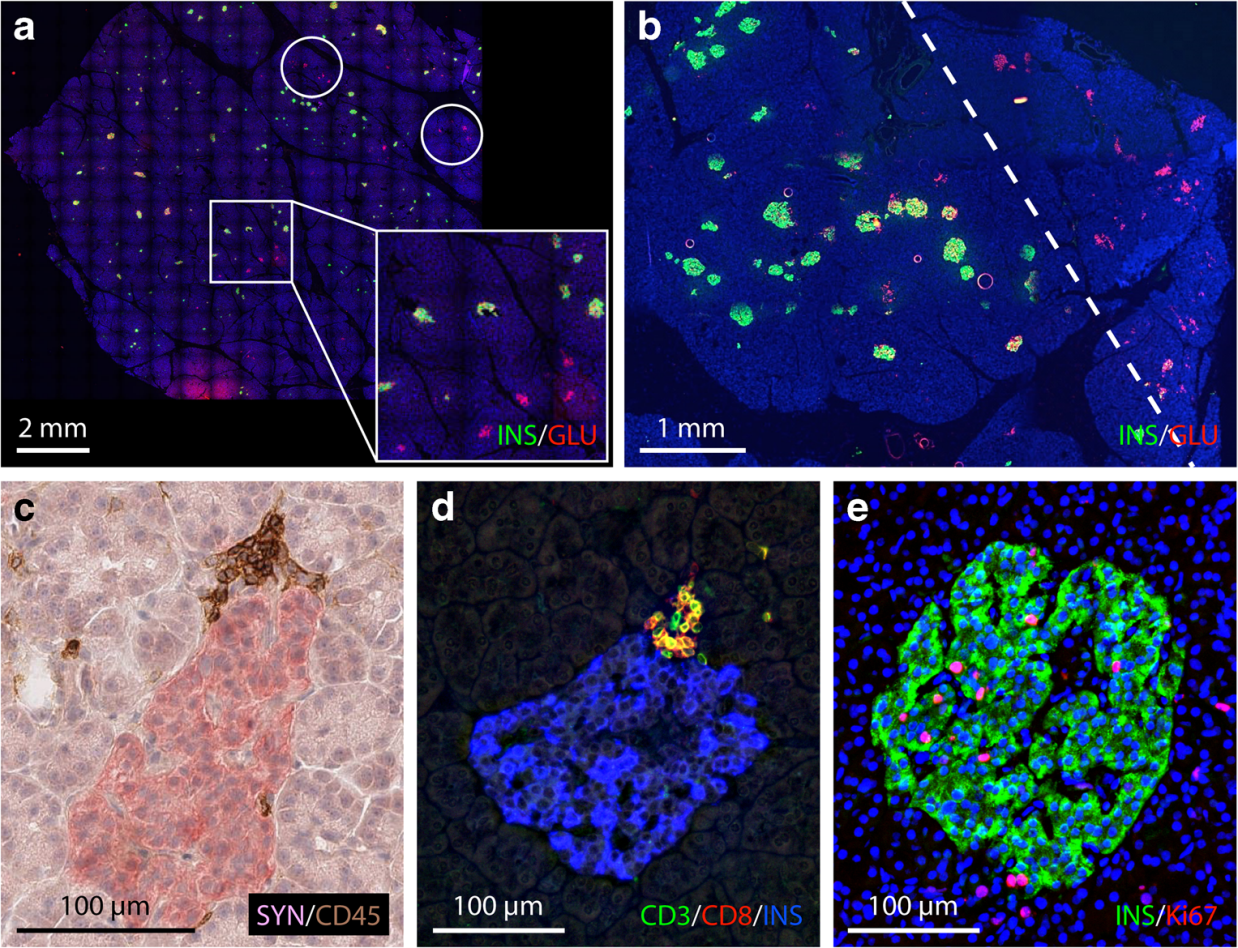
Immunofluorescent staining of pancreas sections from a 17-year-old male organ donor positive for IA-2A and ZnT8 autoantibodies (DBB-A096). Staining for insulin (green) and glucagon (red) shows multiple small foci (**a**) or larger lobular areas of pseudo-atrophic islets (on the right of the dotted line) (**b**). Insulitic lesions were predominantly found at the islet periphery (**c**) and show small lymphocytic infiltrates composed of CD3+CD8+ T-lymphocytes (**d**). Insulin-containing islets show marked positivity for the replication marker Ki67 (**e**)

Donor DBB-A096 involved a 17-year-old male donor who died of severe head trauma, 4 days after admission to ICU. He was tested positive for two autoantibodies (IA-2A, 7.3 WHO units/ml; ZnT8A, 1.6% tracer binding) and had a susceptible HLA-DQA1/DQB1 genotype (*01-05:01/03:01-03:02*) and a HLA class I risk allele (*B15*) [30]. The donor organ consisted of a segmental pancreas of unknown weight, divided into flash frozen samples of approximately 1 cm^3^ without positional information. All tissue samples were available for histopathological analysis. Low magnification tissue sections double-stained for insulin and glucagon showed >30 small focal areas of <2 mm diameter scattered throughout the parenchyma containing 3–5 pseudo-atrophic islets in addition to larger zones of a more lobular character (Fig. 3a–b). Overall, 7.7% of all islets were found to be of a pseudo-atrophic nature. Analysis of multiple biopsies showed insulitis in 0.2% of the islets, with the infiltrate being mainly present in the islet periphery (Fig. 3c). The infiltrates predominantly consisted of CD3+CD8+ T-lymphocytes (65% of all CD3+ cells) and CD3+CD4+ T-lymphocytes (9% of all CD3+ cells) (Fig. 3d). Infiltrates showed a CD20Lo insulitic profile with rare B-lymphocytes and a CD20/CD4 ratio <1.0 [29]. All insulitic islets contained insulin-positive beta cells. A diffuse CD68+ macrophage infiltration was present throughout the pancreatic tissue (data not shown). A beta cell replication rate of 2.8% was found in this donor (Fig. 3e), in addition to a relative beta cell area of 1.33% (Table 2).

## Discussion

Screening for five islet autoantibody types in sera from 556 pancreas donors aged under 25 years without symptomatic type 1 diabetes allowed to identify 25 organs from single marker-positive individuals and two from double positive ones. This substantially expands the limited number of previous single- or multiple autoantibody-positive (*n*=7 and *n*=3, respectively) organ donors under age 25 years and for whom the individual age was reported [3, 4, 19, 21]. This illustrates the considerable effort needed to study pancreas tissue from persons with apparent immune activation against islet cells or, in case of multiple antibody-positivity, who are allegedly in an asymptomatic disease stage [1, 4, 14, 18]. Histopathological alterations were discrete and restricted to both double positive individuals, hereby confirming previous observations [4, 18]. In the present study, histological analysis of the pancreata from both double positive donors showed discrete peri-insulitis in a small fraction of islets, as well as an intact relative beta cell area despite the presence of numerous pseudo-atrophic islets predominantly in a lobular area. A relatively high islet cell replication rate was also noted. An intriguing novel observation is the presence of >30 small focal areas of 3–5 pseudo-atrophic islets scattered throughout the parenchyma of an individual with alleged asymptomatic type 1 diabetes on the basis of positivity for IA-2A, ZnT8A, and the presence of HLA class I- and II-inferred susceptibility [10, 11, 14].

Strengths of the study include the procurement of donor organs, systematic screening for five autoantibody markers, and the use of international consensus criteria to define insulitis. The fact that for most donors only a single usually small tissue sample was received is a limitation. It can thus not be completely excluded that focal histopathological alterations may have been missed in some organs due to the non-random distribution of lesions throughout the organ. Combined screening for both insulitis and pseudo-atrophic islets, as performed in the present study, may decrease but not avoid such a risk. As molecularly defined autoantibodies, including GADA, may contribute to ICA reactivity, joint presence of ICA, and GADA and may not necessarily imply multiple autoantibody positivity [31]. However, high-titer ICA levels, such as present in donor DBB-3504, were associated with increased risk of progression to clinical onset [32]. Likewise in latent autoimmune diabetes in adult patients, the time to insulin-dependence was shorter in joint presence of ICA and GADA than in presence of GADA alone [33]. Part of the ICA positivity in in donor DBB-3504 may have been caused by an autoantibody specificity for which we did not test [13, 23, 34, 35].

When combining our findings with available histological and biological information on multiple autoantibody-positive organ donors (all ages) outside the context of gestational diabetes, polyendocrinopathy or exocrine co-morbidity, and for whom the exact age was reported, it is remarkable that so few histopathological hallmarks of type 1 diabetes were found in the pancreata from these individuals, who are allegedly in an asymptomatic disease stage (Table S3) [3, 4, 18, 20]. Discrete insulitis and foci of pseudo-atrophic islets were observed in 4/4 donors under age 25 years with two or more autoantibody markers and in 2/11 multiple marker-positive donors above that age (Table S3), in line with observations on a higher prevalence of insulitis with younger age at diagnosis in recent-onset type 1 diabetes [2, 36]. The composition of the islet infiltrates was similar in all cases, with a preponderance of CD8+ lymphocytes. The presence of HLA class I alleles reported to confer susceptibility independently of HLA class II-inferred risk [27, 28, 30, 37], and of a high-risk autoantibody profile (IA-2A or ZnT8A plus ≥1 other marker) [11, 13, 34] further support the probability of progressive subclinical disease in (many of) these donors, despite the presence of protective HLA class II haplotypes in some (Table S3); indeed, the latter plays no longer a major role once multiple antibodies have developed [11, 28, 38, 39].

In contrast, neither insulitis nor pseudo-atrophic islets were reported in pancreata from single autoantibody-positive organ donors screened for all antibody markers and reported individually or as a group [3, 4, 18–22]. Conceivably, some of the donors with low-level single autoantibody positivity may represent “statistical” positives due to the use of the 99th percentile of large numbers of healthy controls as cut-off in all assays [23, 24], conferring a risk up to 5% of a false-positive result when testing for five markers. Moreover, single autoantibody positivity is often a low-level transient phenomenon of uncertain significance [40]. Multiple autoantibody positivity, on the other hand, is associated with 90% 20-year risk of progression to clinical onset in children and adults [10, 11, 28].

We found no indication that autoantibody-positivity, be it for one or multiple specificities, was associated with a decreased relative beta cell area in the identified donor organs, in line with previous studies [4, 18, 20, 21, 41, 42]. Interestingly, even in presence of clear insulitic lesions and pseudo-atrophic islets devoid of beta cells, the relative beta cell area was not found to be significantly different from that in matched controls.

Of special interest is donor DBB-A096 with insulitis and multiple small foci of beta cell loss scattered throughout the gland. Such large numbers of small lesions, consisting of 3–5 pseudoatrophic islets, have not previously been described in recent-onset patients, although conversely, a lobular pattern of beta cell survival is relatively common in type 1 diabetes patients, especially those with older age at onset [43–45]. If the small focal lesions are indicative of a pre-diabetic state in this subject, it could be hypothesized that during disease progression, such focal lesions grow in number or size and finally fuse, leading to the characteristic presentation of a pancreatic gland at disease onset with predominantly pseudo-atrophic islets being present, but also with regions in which islets show no histopathological changes. The early disease processes leading to such (multi)focal lesions are unknown. Whether they represent focal points of (auto)immunity with the autoreactive cells migrating to nearby islets, possibly via the connective tissue septa, as observed in mouse models of the disease [46], or represent focal points of (possibly viral) infection spreading outwards [47], can only be speculated.

In the absence of longitudinal studies on islet pathology in high-risk individuals, it cannot be excluded that the observed histopathological changes in multiple autoantibody-positive donor pancreata do not represent true pre-diabetic lesions. This is, however, unlikely since multiple autoantibody-positivity is rarely transient, and confers a high probability of progression to clinically overt disease [11, 28, 38, 39]. The seemingly preserved beta cell mass in the identified multiple autoantibody-positive donors should not be taken as argument against a high probability of later disease progression. Indeed, data by us and others [48, 49] indicate that the functional beta cell mass, as assessed by (stimulated) C-peptide release, only starts to sharply decline within 2 years from diagnosis. The age range of the multiple autoantibody-positive donors in Table S3 indicates an underrepresentation of children in all series of donor pancreata and may point to a selection bias towards slow progressors [50]; hence, the observed histopathological alterations may not be fully representative for asymptomatic childhood-onset type 1 diabetes. The described lesions may also reflect remnants of a low-level immune process during which islets are still able to compensate for any immune-mediated loss as indicated by the presence of islet cell replication in some of these donors. We can also not fully discount the possibility that the relatively minor level of insulitis that is observed is a consequence rather than a cause of the pathogenetic events taking place.

The finding of relatively high levels of replication in the islets of Langerhans of two donors with insulitis is of interest, especially because of the virtual absence of replication in most adult organ donors [51]. However, the interpretation of these observations and the increased CD68 macrophage infiltration in one donor is complicated by studies showing that organ donors with an increased duration of stay in intensive care show evidence of tissue repair, including increased numbers of M2 macrophages, increased vascular density, and increased replication of all pancreatic cell types [52]. As both donors in the present study are characterized by a relatively long duration of stay in intensive care, it cannot be excluded that the increase in beta cell replication found in these two donors is caused by a repair process and is not related to the presumed autoimmune process itself. The percentage of Ki67-positive beta cells was not correlated with relative insulin-positive area, both in our autoantibody-positive and in autoantibody-negative donors (data not shown). This does, however, not exclude compensatory beta cell regeneration via, e.g., transdifferentiation.

In conclusion, evidence for early type 1 diabetes-related histopathological lesions in the pancreas of donors under age 25 years appears restricted to organs from multiple autoantibody-positive individuals at high risk of developing symptomatic disease, in line with observations in older age groups [13]. Donors with single autoantibody-positivity presented no histopathological evidence of immune-mediated beta cell destruction. Single autoantibody-positivity may thus not be a sufficient indicator of islet lesions and not a suitable parameter to select patients for clinical intervention studies. More stringent inclusion criteria are necessary to identify individuals at high risk for the development of type 1 diabetes, such as those proposed in a recent successful immune intervention study where inclusion was limited to relatives of patients with type 1 diabetes with multiple autoantibodies and evidence of dysglycemia [53]. A better insight into the relationship between autoantibody-positivity, insulitis, and beta cell destruction will help in devising better therapies aimed at preventing or curing the disease. However, the fact that none of the donors with multiple autoantibodies and insulitis reported in the present study showed evidence of a decreased relative beta cell area indicates that the relationship between T-cell infiltration and beta cell destruction is more complex than proposed in the classic models of the disease. Alternative models have recently been proposed in which both islet autoimmunity and beta cell dysfunction are suggested to play equally essential roles [54]. Histopathological studies of multiple autoantibody positive donors may help to characterize such pathogenetic pathways, investigating to what extent islet autoimmunity is accompanied by evidence of beta cell dysfunction and stress.

## Supplementary Information

ESM 1 (DOCX 52 kb)

## Data Availability

The detailed datasets generated during and/or analyzed during the current study are available from the corresponding author upon request.

## Abbreviations

GADA: Glutamic acid decarboxylase autoantibodies
IA-2A: Insulinoma-associated protein 2 autoantibodies
IAA: Insulin autoantibodies
ICA: Islet cell cytoplasm autoantibodies
ZnT8A: Zinc transporter 8 autoantibodies
