# Supplementary material for: Insulitis in the pancreas of non-diabetic organ donors under age 25 years with multiple circulating autoantibodies against islet cell antigens
Source: Virchows Arch. 2021 Feb 16;479(2):295–304. doi: 10.1007/s00428-021-03055-z (PMC8364522; doi:10.1007/s00428-021-03055-z)
Supplement: Supplementary file 1 — (DOCX 52 kb) [file 428_2021_3055_MOESM1_ESM.docx]

**Supplementary Material**

**Supplementary methods**

**Screening for insulitis and pseudo-atrophic islets.** Sections were immunohistochemically double-stained with mouse anti-CD45 (clone 2B11+PD7/26; Agilent Technologies; Heverlee; Belgium) and rabbit anti-synaptophysin (Acris; Herford; Germany) (pan-neuroendocrine marker) for the detection of insulitis. Binding was visualized with biotinylated anti-mouse or anti-rabbit IgG (Vector Laboratories; Brulingame; CA; USA) in combination with Vectastain Elite ABC kit and Vectastain ABC-AP kit (Vector Laboratories) using Liquid DAB+ Substrate Chromogen System and Fuchsin+ Substrate Chromogen System (Agilent Technologies) as substrate.

Sections were double-stained for guinea pig anti-insulin and rabbit anti-glucagon (DRC; VUB; Brussels; Belgium) to screen for pseudo-atrophic (insulin-deficient) islets.

**Characterization of leucocytic infiltrates.** Leucocytic infiltrates were immunophenotyped on paraffin sections using immunofluorescent double and triple staining with the following antibodies: rabbit anti-CD3 (Agilent Technologies), mouse anti-CD45 (clone 2B11+PD7/26; Agilent Technologies), mouse anti-CD4 (clone 4B12; Novocastra Reagents; Leica Microsystems; Wetzlar; Germany), mouse anti-CD8 (clone 1A5; Novocastra Reagents), mouse anti-CD20 (clone L26; Agilent Technologies) and mouse anti-CD68 (clone KP1; Agilent Technologies). Binding was visualized with Alexa Fluor 488 anti-rabbit IgG (H+L) or Cy3 anti-mouse IgG (H+L) (all from Jackson ImmunoResearch Laboratories; West Grove; PA; USA) and sections were mounted with fluorescent mounting medium (Agilent Technologies) containing DAPI (10 µg/ml; Sigma-Aldrich; Overijse; Belgium) for DNA staining.

**Quantification of relative beta cell area and beta cell proliferation.** For the quantification of relative beta cell area, 4 µm paraffin sections were immunohistochemically stained with guinea pig anti-insulin. Binding was detected with biotinylated anti-guinea pig IgG in combination with Vectastain Elite ABC kit using Liquid DAB+ Substrate Chromogen System as substrate. Relative insulin-positive cell area was measured according to Rahier et al. [1] on coded slides using a 266 point counting grid at a final magnification of 140x. The number of points on insulin immunoreactive cells (Ni) and pancreatic parenchyma (Np) were counted in 10 randomly chosen microscope fields per case. Relative beta cell area was expressed as percent and calculated as (Ni/Np) x 100. The precision of the point counting technique was evaluated with the formula of Weibel [1]: the calculated relative error in each section was ~11% for a mean relative beta cell area of 1.3%. All morphometric analyses were carried out blinded on coded slides on a minimum of 1cm^2^ of tissue.

Beta cell replication was quantified using double immunofluorescent staining for guinea pig anti-insulin and rabbit anti-Ki67 (1:100; clone SP6; Acris Antibodies GmbH; Herford; Germany). Binding was visualized with DyLight 488 anti-guinea pig IgG (H+L) and Cy3 anti-rabbit IgG (H+L) (all from Jackson ImmunoResearch Laboratories).

**Supplementary tables**

**Table S1.** Positivity for islet autoantibodies in 556 pancreas donors aged 3 months-24 years.

| Type of autoantibody-positivity | Number of donors | Percent of total autoantibody-positive donors |
| --- | --- | --- |
| GADA | 18 | 66.7 |
| ICA | 4 | 14.8 |
| IAA | 3 | 11.1 |
| IA-2A | 0 | 0 |
| ICA + GADA | 1 | 3.7 |
| IA-2A + ZnT8A | 1 | 3.7 |
| Total | **27** | **100** |

**Table S2.** Autoantibody-positive pancreas donors.

| **Donor ID** | **Age** | **Sex** | **BMI** | **ICU** | **Cause of death** | **Ki67+ beta cells** | **Relative beta cell area** | **Autoantibody titer** | | | | |
| --- | --- | --- | --- | --- | --- | --- | --- | --- | --- | --- | --- | --- |
|  | **(yrs)** | **(M/F)** | **(kg/m²)** | **(days)** |  | **(%)** | **(%)** | **ICA** | **IAA** | **GADA** | **IA-2A** | **ZnT8A** |
| DBB-386 | 2 | F | 13.6 | 4 | Anoxia | 0.0 | 1.15 | < | < | **25** | < | < |
| DBB-A130 | 8 | F | 14.8 | 1 | Trauma | 0.0 | 0.85 | < | < | **30** | < | < |
| DBB-2079 | 11 | F | 13.7 | 1 | Other | 0.0 | 1.97 | < | < | **60** | < | < |
| DBB-313 | 13 | M | 23.4 | 2 | Trauma | 0.1 | 2.60 | < | < | **29** | < | < |
| DBB-679 | 13 | M | 15.6 | 1 | Trauma | 0.0 | 1.00 | < | < | **74** | < | < |
| DBB-1495 | 13 | F | 18.4 | 1 | Trauma | 0.0 | 0.69 | < | < | **31** | < | < |
| DBB-A116 | 15 | M | 21.6 | 1 | Trauma | 0.1 | 1.02 | < | < | **49** | < | < |
| DBB-423 | 16 | M | 23.1 | 2 | Trauma | 0.0 | 1.01 | < | < | **43** | < | < |
| DBB-642 | 16 | M | 24.5 | 2 | Trauma | 0.0 | 0.57 | < | < | **71** | < | < |
| DBB-397 | 17 | F | 23.9 | 2 | Cerebrovascular/Stroke | 0.0 | 1.17 | **12** | < | < | < | < |
| DBB-524 | 17 | M | 21.9 | 3 | Trauma | 0.0 | 1.95 | **12** | < | < | < | < |
| DBB-A096 | 17 | M | 21.6 | 4 | Trauma | 2.8 | 1.33 | < | < | < | **7.3** | **1.6** |
| DBB-705 | 18 | M | 19.0 | 7 | Trauma | 0.2 | 2.16 | < | < | **31** | < | < |
| DBB-433 | 19 | M | 24.7 | 6 | Trauma | 0.1 | 1.19 | **12** | < | < | < | < |
| DBB-1512 | 19 | M | 19.9 | 15 | Cerebrovascular/Stroke | 2.3 | 0.80 | < | **1.0** | < | < | < |
| DBB-2171 | 19 | M | 20.6 | 0 | Trauma | 0.0 | 1.65 | < | < | **24** | < | < |
| DBB-421 | 20 | M | 23.9 | 2 | Trauma | 0.0 | 2.13 | < | < | **42** | < | < |
| DBB-1130 | 20 | M | 24.2 | 1 | Trauma | 0.0 | 1.64 | < | < | **141** | < | < |
| DBB-1647 | 20 | M | 21.9 | 40 | Trauma | 0.7 | 0.68 | < | **0.6** | < | < | < |
| DBB-1745 | 20 | M | 22.6 | 8 | Trauma | 0.5 | 1.24 | < | < | **824** | < | < |
| DBB-1856 | 20 | F | 22.0 | 2 | Trauma | 0.0 | 1.70 | < | < | **24** | < | < |
| DBB-2426 | 20 | M | 30.4 | 2 | Trauma | 0.0 | 1.94 | < | < | **56** | < | < |
| DBB-636 | 21 | M | 22.9 | 7 | Trauma | 0.1 | 1.60 | < | **1.0** | < | < | < |
| DBB-1372 | 21 | M | 21.9 | 1 | Cerebrovascular/Stroke | 0.0 | 0.90 | < | < | **35** | < | < |
| DBB-3504 | 22 | M | 26.7 | 11 | Trauma | 0.6 | 1.23 | **400** | < | **29655** | < | < |
| DBB-1237 | 23 | M | 23.1 | 1 | Trauma | 0.0 | 1.18 | < | < | **26** | < | < |
| DBB-2636 | 24 | M | 19.2 | 1 | Trauma | 0.0 | 1.86 | **12** | < | < | < | < |

*ICA is expressed in JDF units, IAA and ZnT8A as % tracer binding and GADA and IA-2A in WHO units/ml. <: autoantibody titer below cut-off value.*

**Table S3.** Demographic and biological characteristics of reported multiple autoantibody-positive organ donors with known age and without gestational diabetes, polyendocrine or exocrine co-morbidity.

| **Donor ID^ref^** | **Age** | **Sex** | **ICA** | **IAA** | **GADA** | **IA-2A** | **ZnT8A** | **HLA** | | **Insulitis** | **PAI** |
| --- | --- | --- | --- | --- | --- | --- | --- | --- | --- | --- | --- |
|  | **(yrs)** |  |  |  |  |  |  | **Class II** | **Class I** |  |  |
| DBBA096^*^ | 17 | M | - | - | - | + | + | S/N | + | 0.2% | 7.7% |
| nPOD6197[2] | 22 | M | na | - | + | + | - | N/N | + | 1.4% | + |
| DBB3504* | 22 | M | + | - | + | - | - | P/N | + | 3.8% | 58.2% |
| nPOD6267[2] | 23 | F | na | - | + | + | - | S/S | + | 6.4% | + |
| nPOD6167[2] | 37 | M | na | - | - | + | + | N/N | - | - | - |
| DBB509[3] | 39 | M | + | - | - | + | - | P/P | + | - | - |
| nPOD6158[2] | 40 | M | - | + | + | - | - | N/N | + | - | - |
| DBB2538[3] | 44 | M | + | - | + | + | + | S/S | + | - | - |
| DBB834[3] | 46 | F | + | na | + | + | na | S/S | + | 2.9% | 3.5% |
| nPOD503[4] | 47 | M | na | - | - | + | + | P/S | na | - | - |
| DBB826[3] | 49 | M | + | - | + | - | - | N/N | + | - | - |
| DBB1203[3] | 50 | F | + | - | + | - | - | P/S | + | - | - |
| DBB1830[3] | 54 | M | + | - | + | - | - | P/S | + | - | - |
| DBB2410[3] | 59 | M | + | + | + | + | + | S/S | + | 8.6% | 1.7% |
| nPOD6080[5] | 69 | F | + | + | + | - | - | N/N | + | - | - |

*PAI: pseudo-atrophic islets; *: this paper; S, N, P: susceptible, neutral and protective DQ or DR haplotype or serotype, respectively; na: not available; + for class I means presence of susceptible risk allele (A*24, A*02, B*39) or serotype (A24, A2, B15)*[6–9]*.*

**Supplementary references**

1. Rahier J, Wallon J, Henquin JC (1981) Cell populations in the endocrine pancreas of human neonates and infants. Diabetologia 20(5):540–546

2. Campbell-Thompson M, Fu A, Kaddis JS, et al (2016) Insulitis and β-cell mass in the natural history of type 1 diabetes. Diabetes 65(3):719–731

3. in ’t Veld P, Lievens D, De Grijse J, et al (2007) Screening for insulitis in adult autoantibody-positive organ donors. Diabetes 56(9):2400–2404

4. Gianani R, Putnam A, Still T, et al (2006) Initial results of screening of nondiabetic organ donors for expression of islet autoantibodies. J Clin Endocrinol Metab 91(5):1855–1861

5. Coppieters KT, Dotta F, Amirian N, et al (2012) Demonstration of islet-autoreactive CD8 T cells in insulitic lesions from recent onset and long-term type 1 diabetes patients. J Exp Med 209(1):51–60

6. Lipponen K, Gombos Z, Kiviniemi M, et al (2010) Effect of HLA class I and class II alleles on progression from autoantibody positivity to overt type 1 diabetes in children with risk-associated class II genotypes. Diabetes 59(12):3253–3256

7. Balke EM, Balti E V, Van Der Auwera B, et al (2018) Accelerated progression to type 1 diabetes in the presence of HLA-A∗24 and-B∗18is restricted to multiple islet autoantibody-positive individuals with distinct HLA-DQ and autoantibody risk profiles. Diabetes Care 41(5):1076–1083

8. Noble JA, Valdes AM (2011) Genetics of the HLA region in the prediction of type 1 diabetes. Curr Diab Rep 11(6):533–542

9. Windsor L, Puschendorf M, Allcock R, et al (2005) Does a central MHC gene in linkage disequilibrium with HLA-DRB1*0401 affect susceptibility to type 1 diabetes? Genes Immun 6(4):298–304
